# Molecular Basis Underlying Common Cutworm Resistance of the Primitive Soybean Landrace Peking

**DOI:** 10.3389/fgene.2020.581917

**Published:** 2020-11-13

**Authors:** Ryu Nakata, Mariko Yano, Susumu Hiraga, Masayoshi Teraishi, Yutaka Okumoto, Naoki Mori, Akito Kaga

**Affiliations:** ^1^Institute of Crop Science, National Agriculture and Food Research Organization, Tsukuba, Japan; ^2^Division of Applied Life Sciences, Graduate School of Agriculture, Kyoto University, Kyoto, Japan; ^3^Division of Agronomy and Horticultural Science, Graduate School of Agriculture, Kyoto University, Kyoto, Japan

**Keywords:** soybean, common cutworm, resistance, chromosome segment substitution lines, Peking

## Abstract

The common cutworm (CCW; *Spodoptera litura*) is one of the major insect pests of soybean in Asia and Oceania. Although quantitative trail loci related to CCW resistance have been introduced into leading soybean cultivars, these do not exhibit sufficient resistance against CCW. Thus, understanding the genetic and metabolic resistance mechanisms of CCW as well as integrating other new resistance genes are required. In this study, we focused on a primitive soybean landrace, Peking, which has retained resistances to various pests. We found a resistance to CCW in Peking by the detached-leaf feeding assay, and subsequently determined the genetic and metabolic basis of the resistance mechanism using chromosome segment substitution lines (CSSLs) of Peking. Several characteristic metabolites for Peking were identified by the metabolomic approach using liquid chromatography/mass spectrometry combined with a principle component analysis. The structure of seven metabolites were determined by nuclear magnetic resonance (NMR) analysis. The genomic segments of Peking on chromosome 06 (Chr06) and Chr20 had a clear association with these metabolites. Moreover, a line possessing a Peking genomic segment on Chr20 inhibited growth of the CCW. The genetic factors and the metabolites on Chr20 in Peking will be useful for understanding mechanisms underlying CCW resistance and breeding resistant soybean cultivars.

## Introduction

The soybean [*Glycine max* (L.) Merr.] is one of the most important leguminous crops in the world and is used for edible proteins, oils, fodder and in various processed foods. The production of soybean ranks fourth to rice, wheat and maize in terms of world crop production. The major production areas are the United States of America, Brazil, Argentina, China, Paraguay, India and Canada. The wild soybean [*G. soja* (Sieb. & Zucc.)], the direct ancestor of the soybean, is distributed mainly in eastern China, eastern Russia, Korea and Japan (Lu, 2004). The domestication of the wild soybean to the soybean is believed to have started from the eleventh century B.C. in China and then spread to surrounding countries (Hymowitz, 1990). Undesirable agricultural traits of the wild soybean, such as seed dormancy, pod dehiscence and elongation of the twining stem, have been removed during the domestication process by farmers. Desirable agricultural traits, such as resistance to biotic and abiotic stress, high yield, and high seed quality, have been selected or introduced into leading soybean cultivars by breeders to ensure stable production under a changing climate and to meet market demand.

Insect pests are one of the factors limiting soybean production. More than 700 species of insect pests are found in soybean (Way, 1994). In Asia and Oceania, the common cutworm [CCW; *Spodoptera litura* (Lepidoptera: Noctuidae)] is one of the major insect pests of soybean. The CCW larvae feed on the leaves of the soybean and cause serious yield losses. The main strategy for controlling CCW is the use of insecticides. The development of CCW-resistant cultivars would reduce insecticide use and stabilize soybean production. In previous studies, two quantitative trait loci (QTLs) for CCW resistance, *CCW1* and *CCW2*, were identified on Chr07 from the CCW-resistant cultivar “Himeshirazu” (Komatsu et al., 2004, 2005). Both QTLs were introduced into a Japanese leading cultivar, “Fukuyutaka” to develop near isogenic lines (NILs), which was confirmed to exhibit significant resistance against CCW (Komatsu et al., 2008), including in a field assessment (Oki et al., 2015). However, the resistance level of NILs harboring *CCW1* and *CCW2* were lower than that of “Himeshirazu” (Komatsu et al., 2008; Oki et al., 2015).

In addition to *CCW1* and *CCW2*, *qRslx1* on Chr07 and *qRslx2* on Chr12 were identified as resistance QTLs from “Himeshirazu” and “Fukuyutaka,” respectively (Oki et al., 2012). These resistance QTLs affected CCW by different mechanisms; *CCW1* and *CCW2* are antibiosis QTLs (i.e., they have adverse effects on insect development and life history) (Komatsu et al., 2005), while *qRslx1* and *qRslx2* are antixenosis QTLs (i.e., they hinder insect behavior) (Oki et al., 2012). Based on these findings, multiple resistance genes would be required to achieve strong and stable resistance against CCW. To find other effective genes that can be introduced into soybean cultivars, the wild soybean has been studied as a source of new resistance genes. Two antixenosis QTLs, *qRslx3* on Chr07 and *qRslx4* on Chr02, were found in a *G. soja* accession [National Agriculture and Food Research Organization (NARO) Genebank accession JP110755]. Another resistant allele was also found in an accession JP267519 (Oki et al., 2019). Since the genetic diversity of the wild soybean is higher than that of the domesticated soybean (Li et al., 2014), it is expected that more effective CCW-resistance genes might be present in wild accessions.

The study of resistance genes often does not enable the explanation of the molecular basis of resistance. A metabolomic analysis is an effective strategy for filling the gap between genotype and phenotype (e.g., resistance). In particular, mass spectrometry (MS) coupled with gas-chromatography (GC) or liquid chromatography (LC) is a powerful technique to evaluate metabolomic diversity within broad genetic plant populations (Luo, 2015; Yandeau-Nelson et al., 2015; Fernie and Tohge, 2017). Many crop phenotypes have been explained by the abundance of primary or secondary metabolites using a metabolomic approach aided with computational and statistical analyses (Hamany Djande et al., 2020).

Among the domestication-related traits, changes in the secondary metabolites are known to occur in 66% of crops (Meyer et al., 2012). For example, toxic or bitter compounds have decreased along with changes in the pigmentation of fruits or seeds. In maize and its wild ancestor, metabolic divergence and candidate genes were investigated (Xu et al., 2019). A large difference in the chemical responses between wild and domesticated wheat cultivars against aphid infection was reported (Batyrshina et al., 2020). A higher amount of organic acids and amino acids were found in the wild soybean than in the cultivated soybean, which are important for the plant to respond to salt stress (Zhang et al., 2016). Therefore, it is expected that chemical compounds for CCW resistance in the wild soybean have decreased or been lost in cultivated soybeans as a result of domestication.

In the present study, we focused on the primitive soybean landrace Peking, which is known to retain resistance to various pests, such as the soybean cyst nematode *Heterodera glycines* (Ross and Brim, 1957), Phytophthora stem and root rot (Jiang et al., 2017) and soybean mosaic virus (Hayes et al., 2000). In addition, Peking is resistant to another noctuid larvae, the corn earworm (*Helicoverpa zea*) (Joshi, 1980); therefore, CCW resistance is also expected. Here, we evaluated CCW resistance and investigated the genetic and metabolic mechanisms of CCW resistance in Peking by using chromosome segment substitution lines (CSSLs) (Watanabe et al., 2018). The CSSLs have partial genomic segments of Peking in a genetic background of the Japanese cultivar Enrei. Thus, an association analysis using the genotype and the metabolite abundance (i.e., phenotype) of CSSLs will enable us to find candidate genes contributing to the metabolite biosynthesis. Although the genetic factors for CCW resistance have been investigated by a genome-wide association analysis (Wang et al., 2015; Liu et al., 2016), the genetic and metabolic background of CCW resistance have not been comprehensively characterized. Understanding the resistance mechanism will help to breed elite cultivars with high CCW resistance.

## Materials and Methods

### Plants and Insects

A total of 103 lines of Peking CSSLs with an Enrei genetic background (Watanabe et al., 2018) and their parents, Enrei and Peking, were used. Seeds were sown in plastic pots containing soil (Vegetable seedling soil type S; Yanmar Co., Ltd., Osaka, Japan) and vermiculite (Vermiculite GS; Nittai Co., Ltd., Osaka, Japan) in a ratio of 1:1 and placed in a greenhouse maintained at ca. 26 ± 8°C under natural light condition [ca. 12 h/12 h (light/dark)]. The CCW larvae were reared on an artificial diet (Insecta-LFS; Nihon Nosan Kogyo Ltd., Yokohama, Japan) under laboratory conditions of 26°C and a 16 h/8 h (light/dark) cycle. The CCW developmental stages were synchronized at each molt by collecting new molting larvae.

### Evaluation of Common Cutworm Resistance by Detached-Leaf Assay

The first and second trifoliate leaves of soybean plants in the V3–5 stages (Fehr and Caviness, 1977) were cut and placed in a petri dish with a ventilation mesh (Insect breeding dish; SPL Life Sciences Co., Ltd., Korea), which were previously lined with moistened filter paper. The 2nd instar CCW larvae (about 5 h after molting) were fasted for 4 h and subsequently placed into the petri dish at 26°C and a 16 h/8 h (light/dark) cycle. Ninety-six hours later, the larvae were frozen in liquid N_2_ and stored at −80°C. After lyophilization, the dry weight of the larvae was measured. In this assay, two CCW larvae were put into one petri dish containing the first and second trifoliate leaves from one soybean plant and one moistened the lined filter paper; four petri dishes were replicated for each line and cultivar. All dishes were piled up to avoid excessive ventilation. Thus, the feeding assay was conducted with eight biological replicates of CCW larvae and four biological replicates of soybean plants.

### Investigation of Characteristic Metabolites for Peking by Liquid Chromatography-Mass Spectrometry

A central leaflet of the first trifoliate leaf of soybean plants in the V3–5 stage was harvested and immersed in an 80% methanol (MeOH) solution (MeOH/H_2_O, v/v) (0.2 mg fresh weight/μL) containing 7-hydroxyflavone (10 ng/μL) as an internal standard. After pulverization and centrifugation at 5,000 *g* for 5 min, the supernatants were filtered through a membrane filter (DISMIC-13HP, 0.45 μm pore size; Toyo Roshi Kaisha, Ltd., Tokyo, Japan). This liquid extraction was conducted with three biological replicates of the Enrei and Peking plants.

The filtrate was analyzed by the Prominence high performance liquid chromatography (HPLC) system coupled with LCMS-2020 (Shimadzu, Co., Kyoto, Japan). The LC separations were performed with an ODS column (Mightysil RP-18GP 50 × 2.0 mm I.D., 5 μm; Kanto Chemical Co., Inc., Tokyo, Japan) at 40°C with 0.2 mL/min flow rate. The solvent program was 5–50% (0–15 min), 50–99% (15–20 min), and 99% (20–25 min) of acetonitrile containing 0.08% acetic acid in H_2_O containing 0.05% acetic acid. The MS was conducted with the following parameters: positive and negative ion mode; scan range, *m/z* 200–1,000; nebulizer gas flow, 1.5 L/min; drying gas flow, 15 L/min; ESI voltage, 4.5 kV in the positive ion mode and −4.5 kV in the negative ion mode; heat block temperature, 200°C; DL temperature, 250°C.

The metabolites in Peking associated with CCW resistance were investigated by comparing the metabolites between Peking and Enrei using a principal component analysis (PCA). The LC-MS data (*m/z* and retention times) was exported using an alignment software (Profiling Solution 1.1 Build 104; Shimadzu, Co., Kyoto, Japan) with the following parameters: retention time, 2–22 min; mass range from 200 to 1,000 Da; ion *m/z* tolerance, 25 mDa; ion retention time tolerance, 1.5 min; ion intensity threshold, 10,000 counts; detecting 20% isomer valley; allowing some ions without isotope peaks. The obtained metabolite array list was analyzed by PCA using SIMCA P (version 13.0.0.0; Umetrics, Umeå, Sweden) after processing by column centering and Pareto scaling.

### Purification of Metabolites A–G From the Peking Leaf Extract

Whole leaves from V5 stage plants of Peking (46 g) were harvested and immersed in 1 L of 80% MeOH solution (MeOH/H_2_O, v/v) at 4°C. After filtration, the filtrate was evaporated under a vacuum to yield a crude extract (4.6 g). This extract was separated with hexane and H_2_O; then, a H_2_O layer (4.5 g extract) was separated with ethyl acetate and H_2_O. The obtained H_2_O layer (3.6 g extract) was passed through an ODS cartridge (Sep-pak C18 (10 g; Waters, Milford, MA, United States) by MeOH elution to remove highly hydrophobic compounds. The eluate (i.e., the through fraction, 3.5 g extract) was collected and separated with a flash chromatograph (CombiFlash Rf + UV; Teledyne ISCO, Lincoln, NE, United States) equipped with a RediSep Rf Gold Reversed-phase C18 column (50 g; Teledyne ISCO, Lincoln, NE, United States). The separation was performed with a 40 L/min flow rate by a stepwise gradient. The solvent program was 30% (0–15 min, Fr. I), 40% (15–30 min, Fr. II), 50% (30–45 min, Fr. III), and 100% (45–70 min, Fr. IV) of MeOH solution (MeOH/H_2_O, v/v). Then, Fr. II (143 g) was chromatographed by HPLC using the Prominence HPLC system. The LC separations were performed with an ODS column (Mightysil RP-18GP 250 × 10 mm I.D., 5 μm; Kanto Chemical Co., Inc., Tokyo, Japan) at 50°C with a 5 mL/min flow rate. The column was eluted in the isocratic mode with a mobile phase containing 31% MeOH (MeOH/H_2_O, v/v) to yield Fr. II-i (*t*_*R*_, 9.6–14.6 min), Fr. II-ii (*t*_*R*_, 14.5–15.9 min), Fr. II-iii (*t*_*R*_, 17.0–20.0 min), Fr. II-iv (*t*_*R*_, 22.4–24.3 min), Fr. II-v (*t*_*R*_, 24.6–26.6 min), Fr. II-vi (*t*_*R*_, 26.8–28.8 min); Fr. II-ii, Fr. II-iv, Fr. II-v, and Fr. II-vi contained metabolites C, E, F, and G in high purity, respectively. Then, Fr. II-i and Fr. II-iii were re-chromatographed using the same apparatus with an ODS column (Mightysil RP-18GP 250 × 4.6 mm I.D., 5 μm; Kanto Chemical Co., Inc., Tokyo, Japan). Then, Fr. II-i was separated in the isocratic mode with a mobile phase containing 16% MeOH (MeOH/H_2_O, v/v) at 60°C with 1 mL/min flow rate to yield Fr. II-i-1 (*t*_*R*_, 58.4–66.2 min) and Fr. II-i-2 (*t*_*R*_, 64.6–69.4 min); Fr. II-iii was separated in the isocratic mode with a mobile phase containing 16% MeOH (MeOH/H_2_O, v/v) at 40°C with 1 mL/min flow rate to yield Fr. II-iii-1 (*t*_*R*_, 16.2–17.6 min). The obtained fraction, Fr. II-i-1, Fr. II-i-2, and Fr. II-iii-1, contained metabolites A, B, and D in high purity, respectively. The purification scheme was summarized in Supplementary Figure S1.

### Structure Determination of Metabolites A–G

The nuclear magnetic resonance (NMR) experiments were performed on a Bruker Avance III 400 spectrometer and Avance III 500 NMR spectrometer (Bruker Corp., Billerica, MA, United States). Each metabolite was dissolved in CD_3_OD (>99.8% D; Euriso-Top, Saint-Aubin, France) containing 0.1% tetramethylsilane as an internal standard. The ^1^H NMR, ^13^C NMR, ^1^H-^1^H COSY, HSQC, and HMBC spectra were measured. The molecular formula and MS/MS fragment of each metabolite were analyzed by LC-Orbitrap MS under the same conditions as described in the next section.

### Liquid Chromatography-Orbitrap Mass Spectrometry Analysis of Metabolites A–G in Chromosome Segment Substitution Lines

A central leaflet of the 1st trifoliate leaf of each line of the CSSLs in the V3 stage was extracted with an 80% MeOH solution (MeOH/H_2_O, v/v) (0.2 mg fresh weight/mL) containing 7-hydroxyflavone (10 μM) as an internal standard. After pulverization and centrifugation at 5,000 *g* for 5 min, the supernatants were filtered through a membrane filter (GL chromato disk 4P, 0.45 μm pore size; GL Sciences Inc., Tokyo, Japan). The leaves of Enrei and Peking were also extracted in the same way. The liquid extraction was conducted with three biological replicates of all plant lines.

The filtrate was analyzed by an Ultimate 3000 SD HPLC system coupled with LTQ Orbitrap discovery (Thermo Fisher Scientific Inc., MA, United States). The LC separations were performed with a PFP column (Ascentis Express 90 Å F5 50 × 2.1 mm I.D., 2.7 μm; Merck KGaA, Darmstadt, Germany) at 50°C with 0.2 mL/min flow rate. The solvent program was 0% (0–1 min), 0–20% (1–21 min), and 99.5% (21–23.5 min) of acetonitrile containing 0.1% formic acid in H_2_O containing 0.1% formic acid. The LTQ Orbitrap MS was operated in the ESI positive ion mode with the following parameters: a capillary temperature of 380°C, sheath gas flow rate of 5 (arbitrary unit), aux gas flow rate of 6 (arbitrary unit), sweep gas flow rate of 0 (arbitrary unit), source voltage of 4.5 kV, capillary voltage of 30 V, and tube lens voltage of 80 V. The MS full scan was acquired in 30,000 resolution at *m/z* 100–1,500 in the FT detector mode. The MS/MS scan was operated by data dependent acquisition (DDA) in 30,000 resolution at *m/z* 100–1,500 in the FT detector mode. For the dynamic exclusion, a repeat count was set at one; the repeat duration and exclusion duration were set at 30 and 20 s, respectively. The most intense ion was selected for the collision induced dissociation (CID) with the following parameters: default charge state, 1; isolation width of *m/z*, 2.0; normalized collision energy, 35; acquisition Q, 0.250; acquisition time, 30 ms; minimum signal required, 1,000. The exact mass of diisooctyl phthalate ([M + H]^+^ = 391.28429) was used for the lock mass.

All collected raw data were imported into the data processing software Compound Discoverer v2.1 (Thermo Fisher Scientific Inc., MA, United States). After retention time alignment and peak picking, the peak area of metabolites A–G and the internal standard were measured.

### Investigation of the Genomic Segments of Peking Associated With the Accumulation of Metabolites A–G and Common Cutworm Resistance

The peak area of each metabolite was divided by that of the internal standard (IS) to give the peak area ratio. In each CSSL and their parents, three peak area ratios derived from three biological replicates were averaged out for each metabolite to represent the amount. The obtained value was used to find the genomic segments that affected the amount of each metabolite by statistical analysis. The genomic segments of the 103 CSSLs were characterized by 321 simple sequence repeat markers that cover all chromosomes evenly (Watanabe et al., 2018). By using the genotype data, the effect of the genotype of each marker on the amounts of each metabolite was evaluated by a one-way ANOVA. The genotype of each marker (i.e., Peking-homozygous, heterozygous, and Enrei-homozygous) was used as a factor, and Type II sum of squares was used for the calculation. Then, the amounts of each metabolite were compared between the lines possessing Peking- and Enrei-genomic segments on each marker.

The CCW resistance of the lines possessing Peking-genomic segments associated with the amounts of each metabolite was evaluated as described in section “Evaluation of Common Cutworm Resistance by Detached-Leaf Assay.”

### Statistics

All statistical analyses were performed with R software (ver. 3.6.1). Boxplots depicted the first quartile minus 1.5 × interquartile range (IQR) (lower whiskers) and the third quartile plus 1.5 × IQR (upper whiskers), the IQR (box) and the median (horizontal line); outliers are indicated by dots outside the whiskers. The quartile was calculated according to Definition 7 in Hyndman and Fan (1996).

## Results

### Common Cutworm Resistance of Peking

**FIGURE 1:**
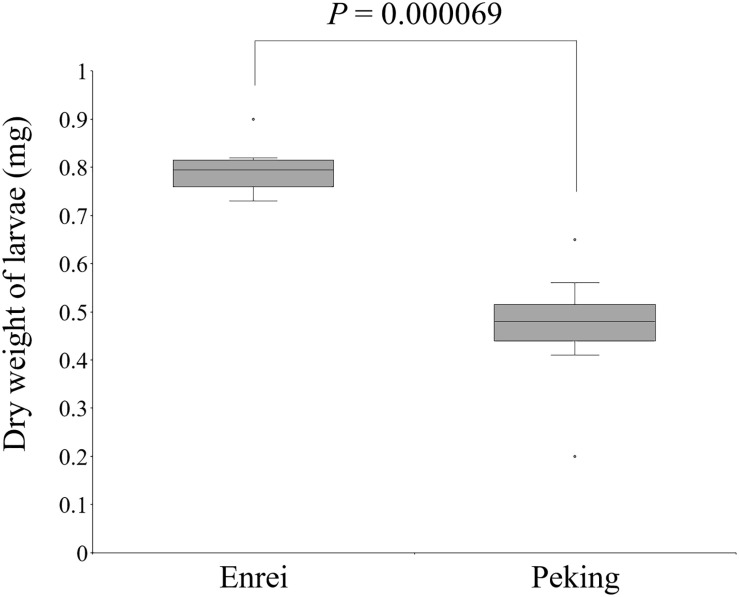
Significant dry weight differences of common cutworm larvae fed on leaves of Enrei and Peking (*n* = 8; Welch’s *t*-test).

The CCW resistance of Enrei and Peking was evaluated by the detached-leaf feeding assay. The weights of the CCW larvae feeding on Peking were significantly lower than those feeding on Enrei (Welch’s *t*-test, *P* < 0.01; Figure 1), suggesting that Peking has factor(s) that inhibit the growth of CCW.

### Investigation of Characteristic Metabolites for Peking by Liquid-Chromatography-Mass Spectrometry

**FIGURE 2:**
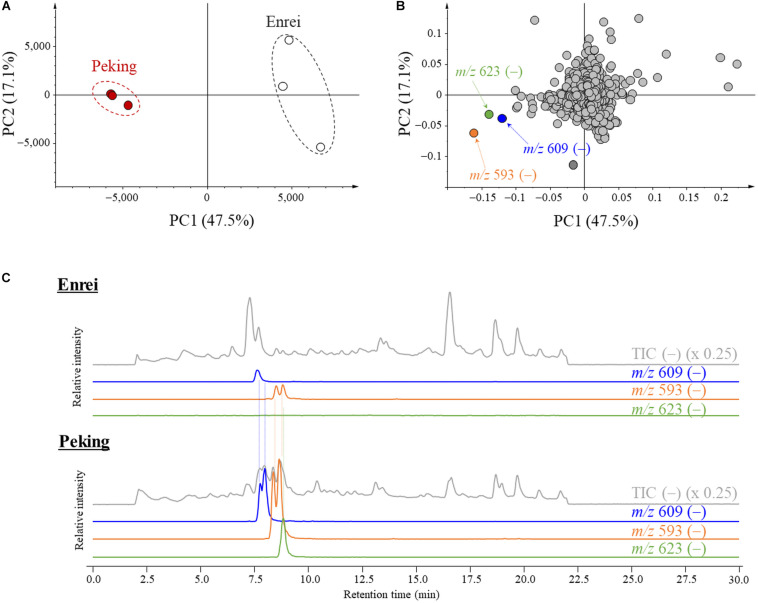
Principal component analysis (PCA) plots and liquid chromatography-mass spectrometry (LC-MS) chromatograms of metabolites isolated from Peking and Enrei. **(A)** PCA score plot. Red circles and white circles indicate Peking and Enrei, respectively (*n* = 3). **(B)** PCA loading plot. Components with large negative loading values are indicated by orange, green and blue circles. The *m/z* value and polarity of each component are described. **(C)** LC-MS chromatograms of Enrei and Peking leaf extracts. Total ion chromatograms (TIC) and extracted ion chromatograms (XIC) are described with the *m/z* value and the fold. Two peaks of *m/z* 609, two peaks of *m/z* 593 and one peak of *m/z* 623 were strongly detected in Peking.

The characteristic metabolites for the leaf of Peking were screened by a comparative metabolic analysis using LC/MS followed by a PCA. The total metabolites were extracted from the leaves of Peking and Enrei, and analyzed by LC-MS. All detected ions were aligned along retention times and *m/z* values, then subjected to the PCA. Score plots exhibited a clear difference of the metabolic profiles between Peking and Enrei (Figure 2A). The principal component (PC) scores of PC1 and PC2 explained 47.5 and 17.1% of the variation, respectively. The PC1 mainly explained the separation between Peking and Enrei; thus, we focused on the PC1 loading value of each component. The components possessing negative loading values indicated that the amounts in Peking were much higher than in Enrei. To find characteristic metabolites for Peking, three components possessing large negative loading values were picked based on PC1 (Figure 2B); these exhibited much larger negative loading values than the other components and represented negative ions at *m/z* 609, 593, and 623 for components 1, 2, and 3, respectively. Further analyses of these ions by LC/MS resolved that a total 5 chromatographic peaks were intermingled: two peaks of *m/z* 609 ions, two peaks of *m/z* 593 ions, and one peak of *m/z* 623 ions (Figure 2C). This is because the poorly separated peaks with the same *m/z* were picked as single components by the alignment software. A total of three ions at *m/z* 609 were further identified in the process of purification (see the next section). Finally, a total of seven characteristic metabolites for Peking were identified and assigned as metabolites A–G.

### Purification and Structural Determination of Metabolites A–G

Metabolites A–G were purified from 46 g of Peking leaves. The purification scheme and yields were summarized in Supplementary Figure S1. Their structures were determined by NMR analysis and LC/Orbitrap-MS spectrometry.

**FIGURE 3:**
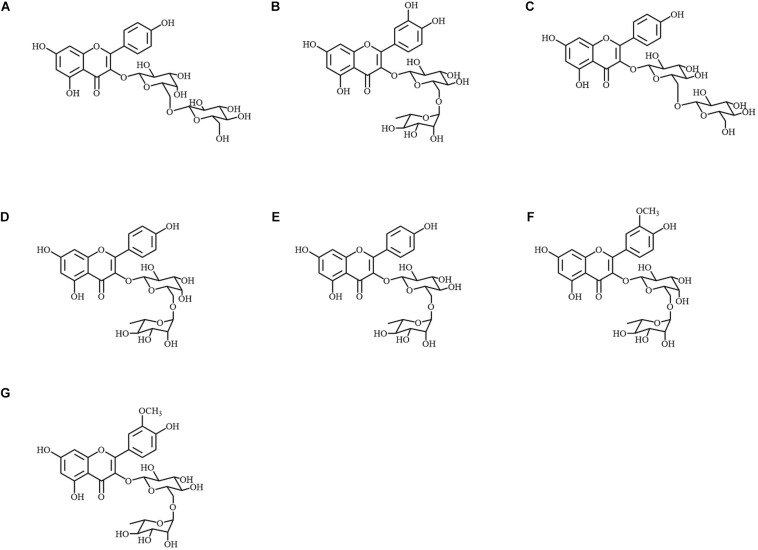
Chemical structures of metabolites A–G isolated from Peking. **(A)** Kaempferol 3-*O*-glucosyl-(1→6)-galactoside; **(B)** quercetin 3-*O*-rutinoside; **(C)** kaempferol 3-*O*-gentiobioside; **(D)** kaempferol 3-*O*-robinobioside; **(E)** kaempferol 3-*O*-rutinoside; **(F)** isorhamnetin 3-*O*-robinobioside; **(G)** isorhamnetin 3-*O*-rutinoside.

Metabolite A was identified as kaempferol 3-*O*-glucosyl-(1→6)-galactoside by 1D and 2D-NMR (Figure 3A). The chemical shifts in ^1^H and ^13^C-NMR, and the correlation observed in ^1^H-^1^H COSY and HMBC are summarized in Supplementary Table S1. The position at which the sugars conjugated were determined by the HMBC correlation between H-1″ and C-3, and also between H-1^″′^ and C-6″. The spectrum of ^1^H-NMR was also in accordance with values in the literature (Tanaka et al., 2001). Then, the molecular formula was confirmed by MS spectrometry as C_27_H_31_O_16_ (error: −1.25 ppm, Supplementary Table S2). In the MS/MS analysis, the diglycoside moiety was also signified by two glycoside-specific fragments, [M + H − C_6_H_10_O_5_]^+^ and [M + H − C_12_H_20_O_10_]^+^ ions.

Metabolites B–G were identified as quercetin 3-*O*-rutinoside, kaempferol 3-*O*-gentiobioside, kaempferol 3-*O*-robinobioside, kaempferol 3-*O*-rutinoside, isorhamnetin 3-*O*-robinobioside and isorhamnetin 3-*O*-rutinoside, respectively (Figures 3B–G). The observed chemical shifts accorded with those of values reported in the literature (Demırezer et al., 2006; Güvenalp et al., 2006; Nogueira and Lopes, 2012; Duong et al., 2017; Viet Thanh et al., 2018; Supplementary Table S3). Each molecular formula and the MS/MS fragments are summarized in Supplementary Table S2.

### Genomic Segments of Peking Associated With the Accumulation of Metabolites A–G and Common Cutworm Resistance

**FIGURE 4:**
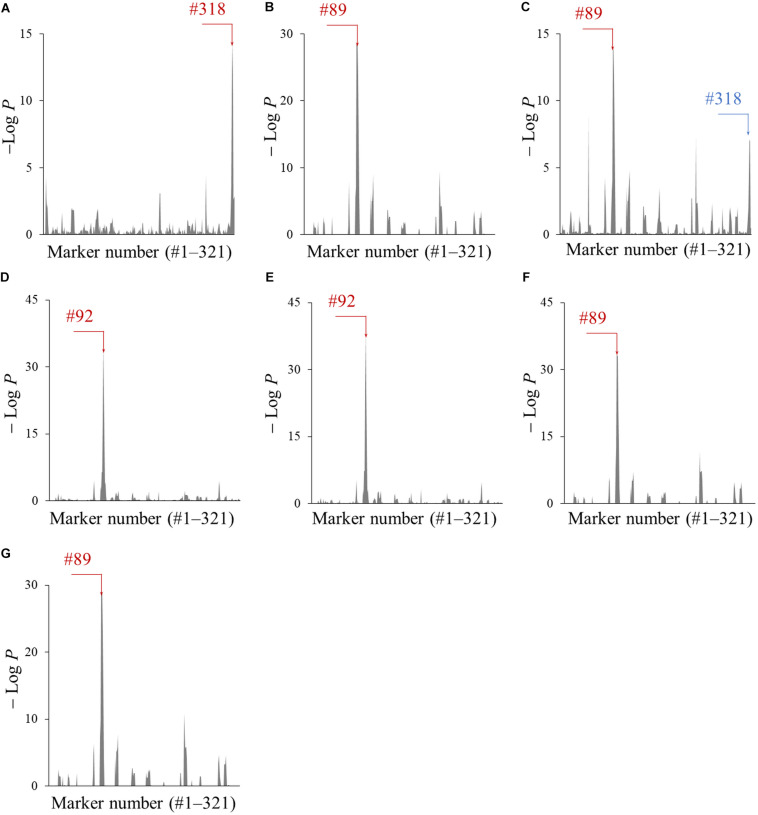
The association of genomic segments of Peking with the amounts of metabolites A–G **(A–G)**. The −Log*P* values obtained by the ANOVA was plotted against the corresponding 321 markers. The markers with the largest − Log*P* values are indicated with red arrows and the marker number (#). The marker which was associated with amounts of metabolite C is indicated by a blue arrow and the marker number (#).

The genomic segments of Peking associated with the accumulation of metabolites A–G were screened using the CSSLs. The amounts of each metabolite in all 103 CSSLs were quantified by LC-Orbitrap MS equipped with a PFP column, which enabled the separation of metabolites A–G in a short run time (Supplementary Figure S2). Next, the association of 321 marker genotypes with the amount of metabolites A–G was tested by a one-way ANOVA. The obtained *P*-values, converted to − Log*P*, were plotted with respect to each marker number (#) (Figure 4). A large − Log*P* value in these plots indicated that the genomic segments of Peking tagged by the marker affected the amount of each metabolite. The marker numbers (#) with the largest − Log*P* values were #318 (metabolite A), #89 (B), #89 (C), #92 (D), #92 (E), #89 (F), and #89 (G) (indicated by red colored arrows in Figure 4). The marker #89 and #92 were located on Chr06, while the marker #318 was on Chr20.

**FIGURE 5:**
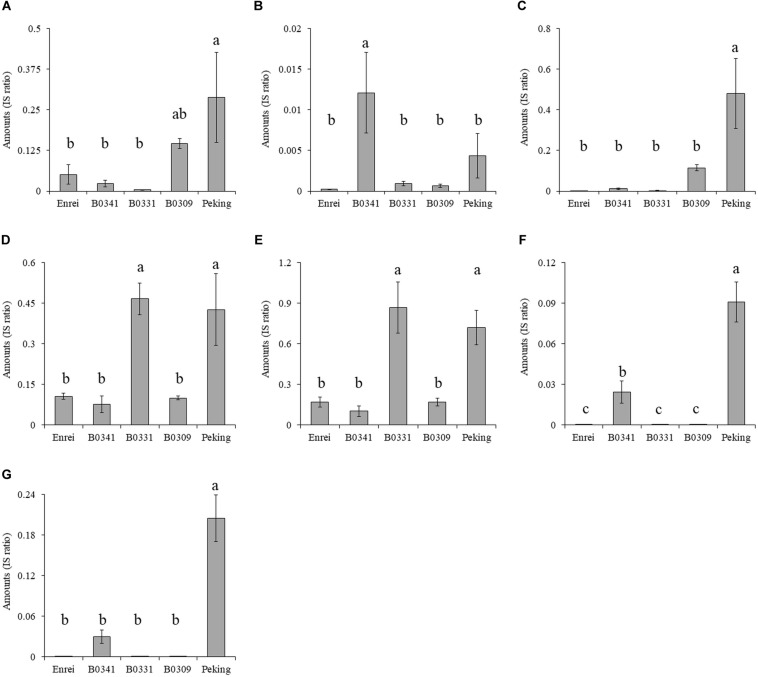
The amounts of metabolites **(A–G)** in three chromosome segment substitution lines (B0341, B0331 and B0309), Enrei and Peking. Different letters indicate significant differences between lines (Tukey’s test, *n* = 3, *P* < 0.05). B0341, B0331 and B0309 possess the Peking-genomic segment tagged by the markers #89, #92 and #318, respectively.

The amounts of metabolites A–G in three CSSLs (B0341, B0331, and B0309), Enrei and Peking were compared (Figure 5). The CSSLs B0341, B0331 and B0309 had either one of the Peking-genomic segments tagged by the marker #89, #92, and #318, respectively. The amount of metabolite A in B0309 was higher than that in the other two lines and Enrei. The CSSL B0341 contained a higher amount of metabolites B, F, and G than the other two lines and Enrei, while B0331 contained a higher amount of metabolites D and E. In addition, the amount of metabolites C in B0309 was slightly higher than that in the other two lines and Enrei. According to these results, the amount of metabolite A, B, D, E, F, and G was clearly increased by the presence of the Peking-genomic segment, which exhibited the largest − Log*P* values in the ANOVA for each metabolite (Figure 4). On the other hand, the amount of metabolite C was not increased by this (i.e., the marker #89) but increased by the marker #318, which exhibited a relatively large − Log*P* value in the ANOVA (see the blue colored arrow in Figure 4C).

**FIGURE 6:**
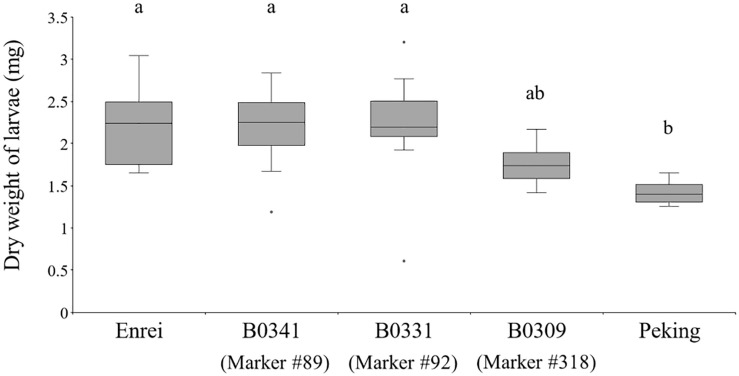
The dry weight of common cutworm larvae fed on leaves of the three chromosome segment substitution lines (B0341, B0331, and B0309), Enrei and Peking. Different letters indicate significant differences between lines (Tukey’s test, *n* = 8, *P* < 0.05).

The effect of the Peking-genomic segments tagged by the markers #89, 92, and 318 on CCW growth was examined (Figure 6). Among the three CSSLs (B0341, B0331, and B0309), Enrei and Peking, the weight of larvae fed on B0309 was smaller but not significantly different from that of larvae fed on Enrei and the other CSSLs. Although B0309 did not show the same level of CCW resistance of Peking, the Peking-genomic segments tagged by the marker #318 have genetic factor (s) that inhibit the growth of CCW. In the vicinity of the marker #318 (Chr20: 40,999,771 bp), the biosynthetic genes or pseudogenes of the biosynthesis of metabolite A and C were not found on the Glyma.Wm.82.a2.v1 assembly using SoyBase (last accessed on 24 June 2020)^1^. However, a glycosyltransferase gene, Glyma.20g167900, was identified on Chr20: 40,568,843–40,578,286 bp. This gene is annotated to have quercetin 3-*O*-glucosyltransferase activity (GO:0080043) and quercetin 7-*O*-glucosyltransferase activity (GO:0080044). In the vicinity of marker #89 (Chr06: 18,737,344 bp), flavonoid 3′-monooxygenase (Glyma.06g202300) is located on Chr06: 18,731,105–18,738,025 bp. In the vicinity of marker #92 (Chr06: 48,016,322 bp), the biosynthetic genes of these metabolites were not identified; however, a glycosyltransferase gene, Glyma.06g285700, is located on Chr06: 47,431,347–47,433,567 bp, 584,975 bp apart from the marker #92. This gene is annotated to have quercetin 3-*O*-glucosyltransferase activity (GO:0080043) and quercetin 7-*O*-glucosyltransferase activity (GO:0080044).

## Discussion

In the present study, we revealed the genetic and metabolic background of CCW resistance of Peking using CSSLs. Seven characteristic metabolites for Peking expected to associate with CCW resistance were identified by metabolomic analyses using LC-MS followed by a PCA. Two Peking genomic segments on Chr06 and Chr20 were associated with the accumulation of these metabolites. Subsequently, the Peking genomic segment on Chr20 was associated with CCW resistance.

The CSSL B0309 possessing the Peking Chr20 genomic segment tagged by marker #318 inhibited the growth of CCW (Figure 6) and accumulated metabolite A (kaempferol 3-*O*-glucosyl-(1→6)-galactoside) and C (kaempferol 3-*O*-gentiobioside) (Figure 5). These results suggest that metabolite A and C are the potential resistance factors against CCW. The biological activity of these metabolites on insects have not been reported; however, Varghese et al. (2013) revealed α-glucosidase inhibitory activity of metabolite C. α-Glucosidase is one of the important digestive enzymes in the insect midgut (Terra et al., 2012). The inhibition of α-glucosidase reduces the survival and development of insects, including CCW larvae (Kaur et al., 2019). To demonstrate that metabolite A and C are resistance factors in Peking, the evaluation of the growth inhibition of CCW larvae and their biological activity using purified substances is ongoing. Although genes related to the biosynthesis and accumulation of metabolite A and C have not been fully identified, the biosynthesis of kaempferol (i.e., an aglycone moiety) following glycosylation is deduced. In the vicinity of the marker #318, two genes annotated as quercetin 3-*O*-glucosyltransferase activity and quercetin 7-*O*-glucosyltransferase. Further functional studies of this gene and its relationship with CCW resistance is required. The comprehensive gene expression study based on RNA-seq analysis may facilitate narrow down candidate genes in the vicinity of the marker #318. In addition, genes in other segments are also required for reproducing the same level of CCW resistance as in Peking, because this segment alone did not confer the high accumulation level of metabolites A and C that was observed in Peking (Figure 5) and did not produce strong CCW resistance comparable to that of Peking (Figure 6).

The metabolites B (quercetin 3-*O*-rutinoside), F (isorhamnetin 3-*O*-robinobioside), and G (isorhamnetin 3-*O*-rutinoside) were accumulated in B0341 possessing the marker #89 (Figure 5). In the vicinity of marker #89, flavonoid 3′-monooxygenase is located on Chr06. Flavonoid 3′-monooxygenase is responsible for the biosynthesis of the aglycone of metabolites B, F, and G. This enzyme converts kaempferol to quercetin by oxidation at position 3′, which is an aglycone of metabolite B; subsequently, quercetin is converted to isorhamnetin by methyltransferase, which is an aglycone of metabolite G. The accumulation level of metabolite B in B0341 was clearly higher than that in Peking, whereas that of metabolites F and G were lower than in Peking. The over-accumulation of metabolite B in B0341 would be caused by a lack of other Peking-genomic segments, which would precede the metabolism of metabolite B to others in Peking. In contrast, the low accumulation level of metabolite F and G would be due to the requirements of other Peking-genomic segments to reproduce the high accumulation levels, as observed in Peking.

The accumulation level of metabolites D (kaempferol 3-*O*-robinobioside) and E (kaempferol 3-*O*-rutinoside) in B0331 were the same as in Peking (Figure 5). This suggests that the Chr06 Peking-genomic segment tagged by the marker #92 contained gene(s) that enable the accumulation of these metabolites at the same levels as observed in Peking. The biosynthetic genes of these metabolites were not identified in the vicinity of marker #92, while two genes (quercetin 3-*O*-glucosyltransferase activity and quercetin 7-*O*-glucosyltransferase activity) were located 585 kbp apart from the marker #92.

Although B0309 inhibited the larval growth of CCW, the inhibitory effect was not at the same level as Peking (Figure 6). This indicates that other Peking-genomic segments would be required to reproduce the same resistance level of Peking. In previous studies, two antibiosis QTLs (Komatsu et al., 2005) and one antixenosis QTL (Oki et al., 2012) for CCW from a resistant cultivar, “Himeshirazu,” were reported. Among them, two antibiosis QTLs, *CCW1*, and *CCW2*, did not reproduce CCW resistance at the same level as “Himeshirazu” (Komatsu et al., 2004). Although there are limitations for evaluating CCW resistance in terms of the replication scale and uniformity of plant and CCW growth, we are planning to evaluate abundance of characteristic secondary metabolites for Peking and the larval growth of CCW for all the CSSLs to advance our understanding of the resistance mechanisms of Peking.

In the present study, we uncovered CCW-resistant genetic factor(s) on Chr20 derived from the primitive soybean landrace Peking. In addition, metabolite A (kaempferol 3-*O*-glucosyl-(1→6)-galactoside) and C (kaempferol 3-*O*-gentiobioside) are potential resistance compounds. These would be novel resistant factors because *CCW1* and *CCW2* in “Himeshirazu” were identified on Chr07 (Komatsu et al., 2005). On the other hand, a direct evidence for CCW resistance of metabolite A and C is required for proving the contribution of these metabolites to CCW resistance in Peking. Thus, we are planning to evaluate CCW resistant activity of these metabolites by feeding assay using purified substances. In the present soybean breeding program in Japan, efforts of pyramiding several CCW-resistant genes from wild soybeans have continued (Oki et al., 2017). Although the QTLs related to CCW resistance found in “Himeshirazu” (*CCW1* and *CCW2*) have been introduced into a leading cultivar, it has not exhibited strong enough resistance to CCW. Therefore, the genetic factors on Chr20 will contribute to understanding CCW resistance mechanisms and can be used to improve the resistance of current resistant cultivars in a breeding program. Since break down linkage between genetic factors for resistant and undesirable characters and integration of resistant genetic factors into genetic background of a leading cultivar are required, multigenic nature of resistance will impedes breeding within short period even if we found good wild or un-adapted genetic resources for the resistance. Similarly, the primitive landrace Peking may have undesirable characters beside blackish and flat small seed. CSSLs used in the present study are useful to examine undesirable characters and will be good materials for the development of a durable CCW-resistant cultivars. Especially, a series of CSSLs retain all resistance factors of Peking because CSSLs cover all chromosomes in contrast to breeding materials which lose many minor resistant factors during selection in the breeding. If we can develop methods that efficiently evaluate metabolites that collectively affect the insect resistance as shown in the present study and evaluate precisely morphological traits such as shape of trichome to the insect resistance, an elite cultivar with more durable insect resistance can be developed by inter-crossing CSSLs without undesirable characters in future.

## Data Availability Statement

All datasets generated for this study are included in the article/Supplementary Material. Metabolomics data have been deposited to the EMBL-EBI MetaboLights database (Haug et al., 2020) with the identifier MTBLS1960. The complete dataset can be accessed here https://www.ebi.ac.uk/metabolights/MTBLS1960.

## Author Contributions

RN, MY, MT, YO, NM, and AK: study design and data analysis. RN and MY: phenotype data collection. RN, MY, and SH: metabolomics data collection. MY and NM: structural determination of metabolites. RN, NM, and AK: manuscript drafting. All authors contributed to the article and approved the submitted version.

## Conflict of Interest

The authors declare that the research was conducted in the absence of any commercial or financial relationships that could be construed as a potential conflict of interest.

## Footnotes

1 https://soybase.org/

## Supplementary Material

The purification scheme and LC-MS chromatograms (XIC) of authentic samples of the metabolites are provided in Supplementary Figures S1, S2. The NMR spectra of metabolite A is presented as Supplementary Table S1. The molecular formulae and MS/MS fragments obtained by LC/Orbitrap-MS analyses are summarized in Supplementary Table S2. The chemical shifts of the metabolites are provided in Supplementary Table S3.
