# Supplementary material for: Molecular Basis Underlying Common Cutworm Resistance of the Primitive Soybean Landrace Peking
Source: Front Genet. 2020 Nov 13;11:581917. doi: 10.3389/fgene.2020.581917 (PMC7693442; doi:10.3389/fgene.2020.581917)

Supplementary Material

**Molecular basis underlying common cutworm resistance of the primitive soybean landrace Peking**

**Ryu Nakata^1^****^†^, Mariko Yano^2^, Susumu Hiraga^1^, Masayoshi Teraishi^3^, Yutaka Okumoto^3‡^, Naoki Mori^2^, Akito Kaga^1^***

^1^ Institute of Crop Science, National Agriculture and Food Research Organization, Tsukuba, Japan

^2^ Division of Applied Life Science, Graduate School of Agriculture, Kyoto University, Kyoto, Japan

^3^ Division of Agronomy and Horticultural Science, Graduate School of Agriculture, Kyoto University, Kyoto, Japan

^†^ Present address: Department of Bioscience and Biotechnology, Kyoto University of Advanced Science, Kameoka, Japan

**^‡^** Present address: Department of Agricultural Science and Technology, Setsunan University, Hirakata, Japan

*** Correspondence:**Akito Kaga
[kaga@affrc.go.jp](mailto:kaga@affrc.go.jp)

# Supplementary Data

The purification scheme and LC-MS chromatograms (XIC) of authentic samples of the metabolites are provided in Supplemental Fig. S1 and Fig. S2. The NMR spectra of metabolite A is presented as Table S1. The molecular formulae and MS/MS fragments obtained by LC/Orbitrap-MS analyses are summarized in Supplemental Table S2. The chemical shifts of the metabolites are provided in Supplemental Table S3.

# Supplementary Figures and Tables

## Supplementary Figures


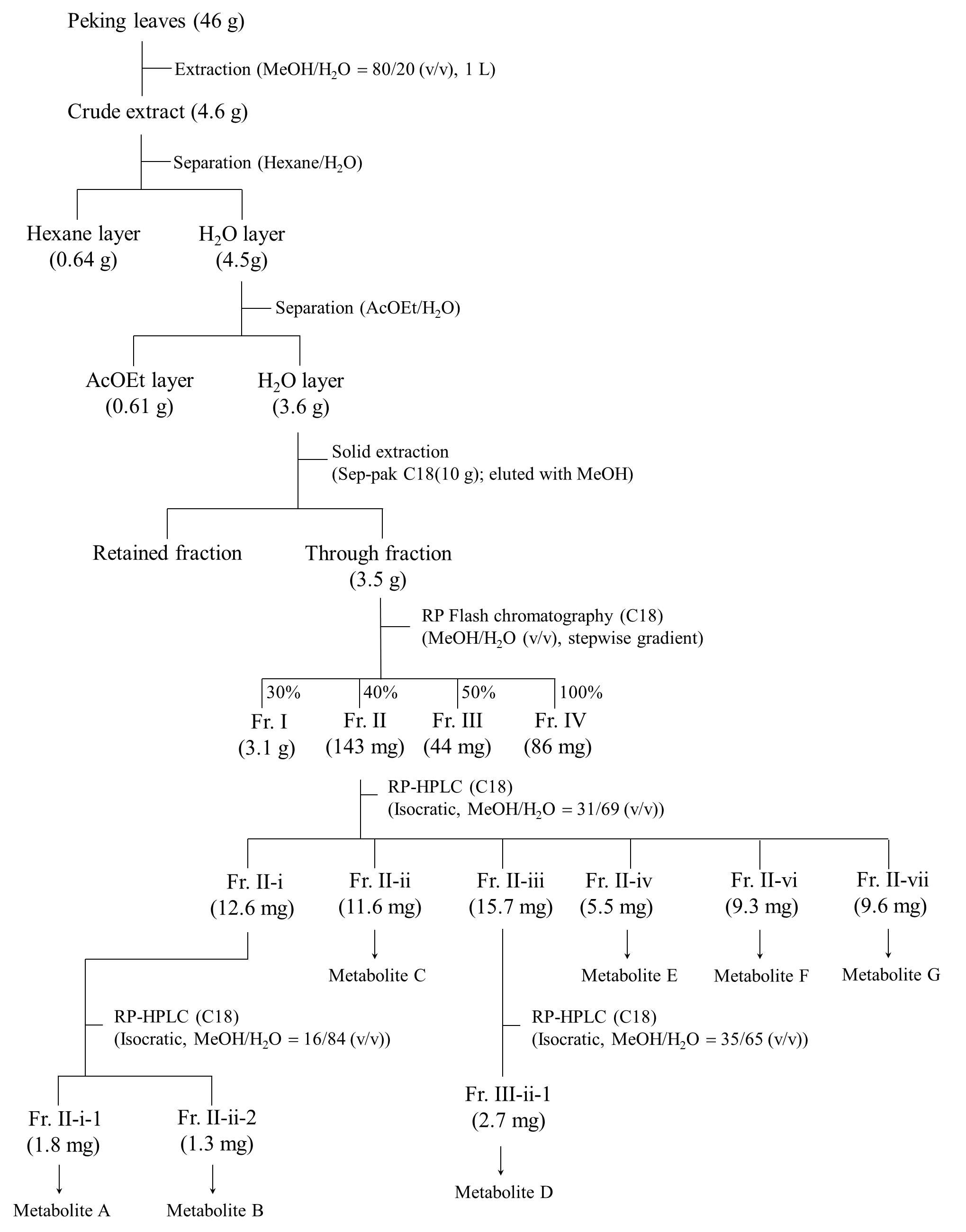


**Supplemental Figure 1.** Metabolite purification scheme from Peking leaves.

**
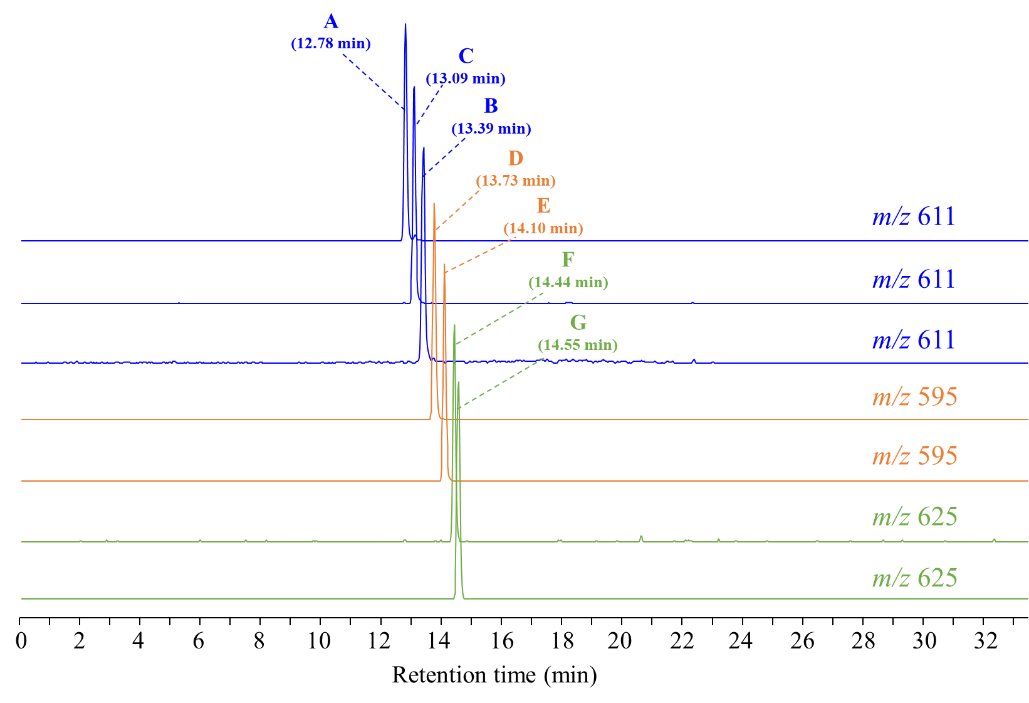
**

**Supplemental Figure 2.** Liquid chromatography-mass spectrometry chromatograms (extracted ion chromatograms) of authentic samples of metabolites A–G. A PFP column enabled the clear separation of all peaks.

## Supplementary Tables

Supplemental Table 1. Nuclear magnetic resonance spectra of metabolite A isolated from Peking soybean.

**
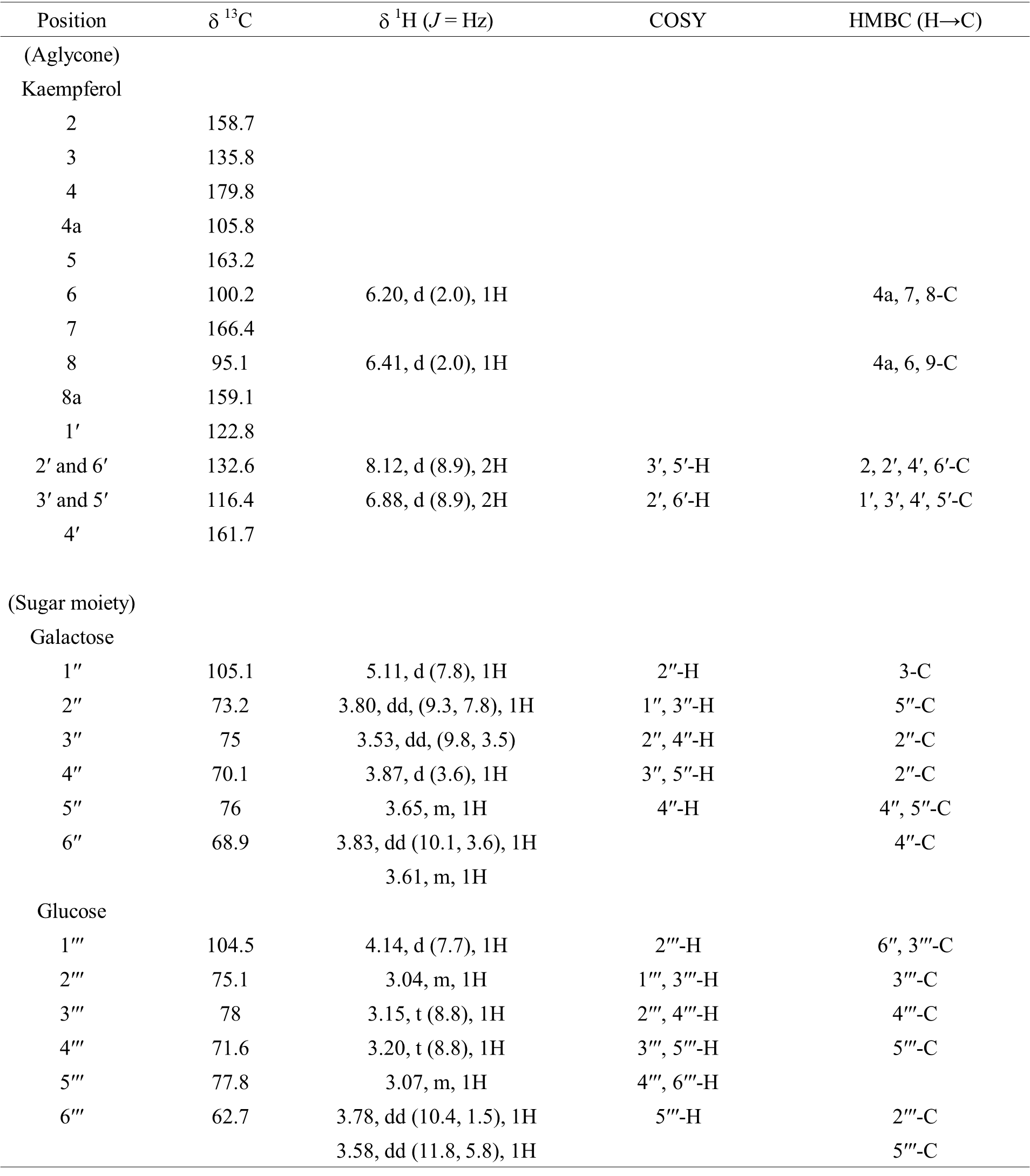
**

Supplemental Table 2. Molecular formulae and tandem mass spectrometry (MS/MS) fragments obtained by liquid chromatography/Orbitrap-MS analyses.

**
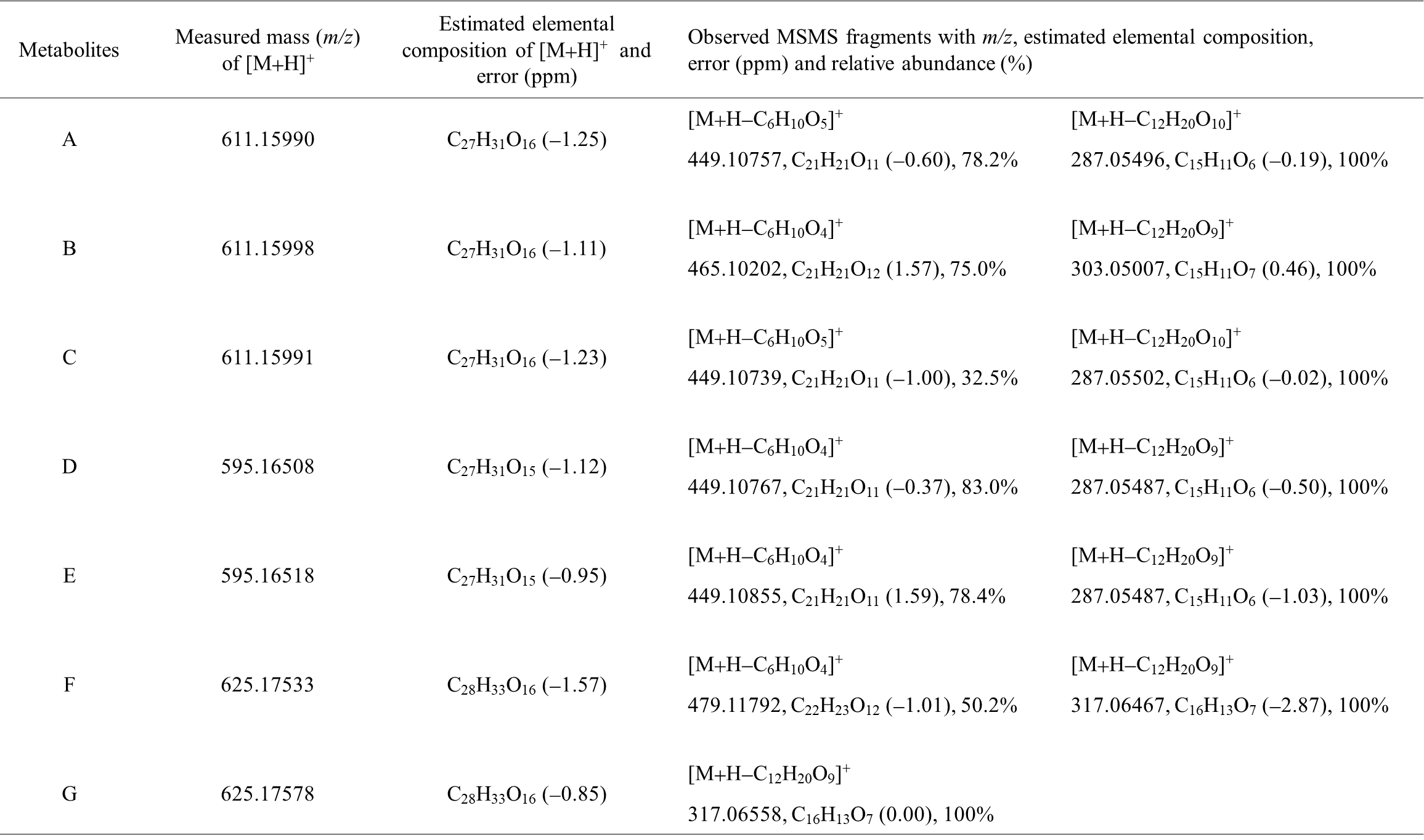
**

Supplemental Table 3. ^13^C Chemical shifts of metabolites B–G isolated from Peking.


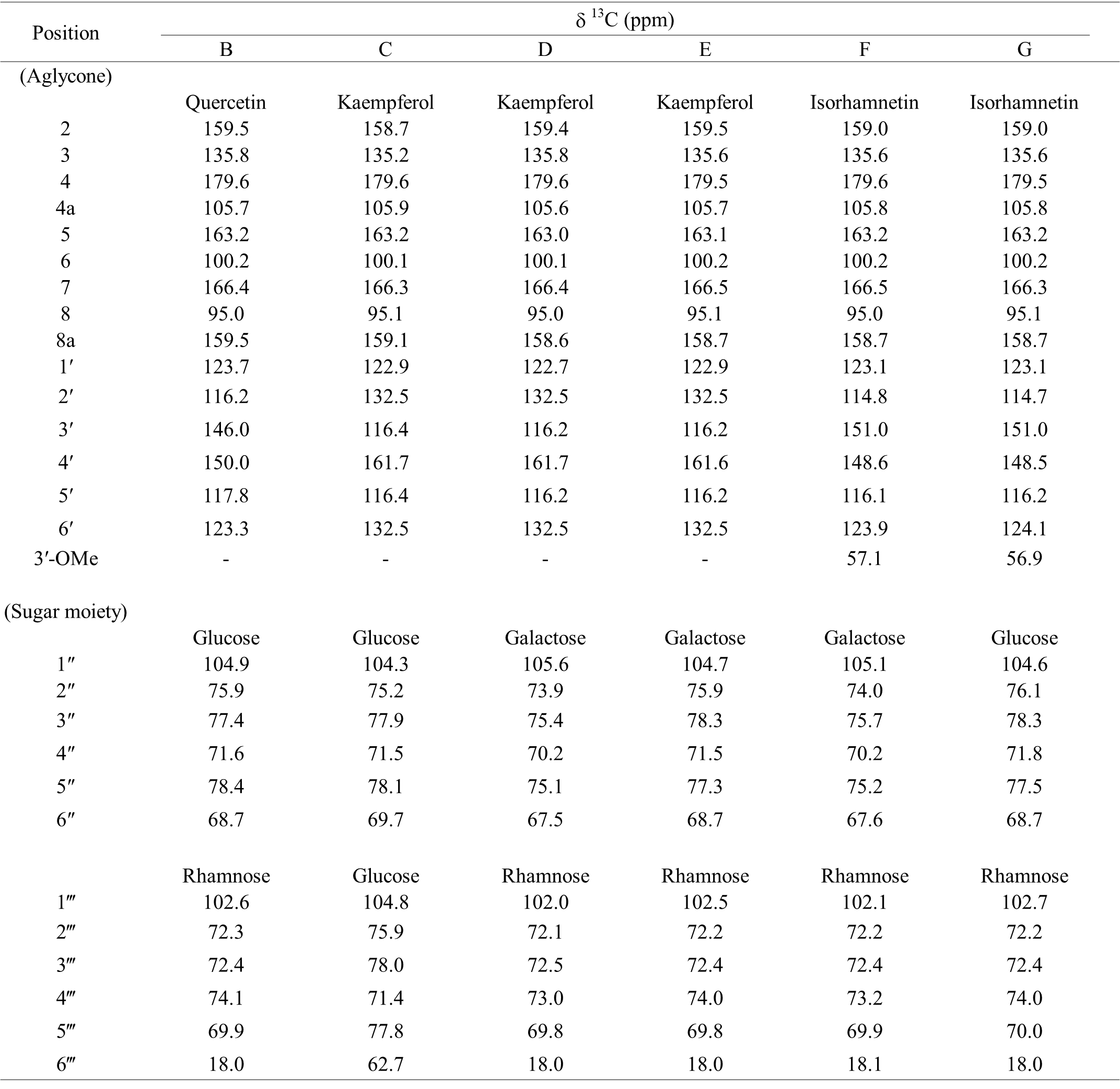

Supplement: Supplementary file 1 [file Data_Sheet_1.docx]
